# The Growing Medical Need for Tracheal Replacement: Reconstructive Strategies Should Overcome Their Limits

**DOI:** 10.3389/fbioe.2022.846632

**Published:** 2022-05-09

**Authors:** Davide Adamo, Giulia Galaverni, Vincenzo Giuseppe Genna, Filippo Lococo, Graziella Pellegrini

**Affiliations:** ^1^ Interdepartmental Centre for Regenerative Medicine “Stefano Ferrari”, University of Modena and Reggio Emilia, Modena, Italy; ^2^ Holostem Terapie Avanzate S.r.l., Modena, Italy; ^3^ Università Cattolica del Sacro Cuore, Rome, Italy; ^4^ Thoracic Surgery Unit, Fondazione Policlinico Universitario A. Gemelli IRCCS, Rome, Italy

**Keywords:** tracheal replacement, tracheal surgeries, clinical outcomes, preclinical studies, regenerative medicine, tissue engineering, allotransplantation

## Abstract

Breathing, being predominantly an automatic action, is often taken for granted. However, respiratory diseases affect millions of people globally, emerging as one of the major causes of disability and death overall. Among the respiratory dysfunctions, tracheal alterations have always represented a primary challenge for clinicians, biologists, and engineers. Indeed, in the case of wide structural alterations involving more than 50% of the tracheal length in adults or 30% in children, the available medical treatments are ineffective or inapplicable. So far, a plethora of reconstructive approaches have been proposed and clinically applied to face this growing, unmet medical need. Unfortunately, none of them has become a well-established and routinely applied clinical procedure to date. This review summarizes the main clinical reconstructive attempts and classifies them as non-tissue engineering and tissue engineering strategies. The analysis of the achievements and the main difficulties that still hinder this field, together with the evaluation of the forefront preclinical experiences in tracheal repair/replacement, is functional to promote a safer and more effective clinical translation in the near future.

## Introduction

Tracheal and main bronchi dysfunctions represent an unmet medical need in respiratory medicine. Indeed, congenital malformations, malignancies, inflammations, infections, or even traumatic events, including postoperative complications, can alter tracheal and main bronchi structure and function, heavily affecting patients’ life. Nowadays, this issue represents an extraordinary leading-edge topic. Indeed, as recently stated by the European Laryngological Society, the heightened number of long-term intubations and the huge tracheostomy rate in critically ill COVID-19 patients might shortly determine an unprecedented increase in laryngotracheal granulomas, stenosis, malacia, tracheal necrosis, tracheo-oesophageal and trachea-innominate fistulae (Alturk et al., 2020; Mattioli et al., 2021; Piazza et al., 2021). The first-choice treatment for managing short tracheal structural alterations is based on tracheal resection and reconstruction by end-to-end anastomosis. However, surgery is inapplicable in longer tracheal injuries exceeding 5 cm in adults and 3 cm in children, and only palliative care can be offered to these patients (Delaere et al., 2019).

To date, a variety of approaches has been proposed and clinically adopted to replace long-segment tracheal defects and reconstruct this vital organ. However, despite the great efforts, the limited results clearly pointed out that a final solution for restoring a functional respiratory system is extremely challenging and far from being addressed (Grillo, 2002; Soriano et al., 2021).

### Why is the Trachea so Hardly Replaceable?

Despite its apparently simple shape and structure, the trachea is a complex organ, challenging to be replaced. Indeed, it is a semiflexible tube measuring approximately 5 cm in children under 3 months of age (Lee and Yang, 2001) and reaching 10–13 cm in adults (Cinar et al., 2016). It comprises up to 22 C-shaped hyaline cartilaginous rings posteriorly connected by smooth muscle embedded into a fibro-elastic connective tissue (Brand-Saberi and Schäfer, 2014). This structure gives a peculiar lateral rigidity and longitudinal flexibility, critical for controlling the trachea lumen diameter, preventing its collapse and supporting the trachea during inspiration/expiration (Grillo, 2002; Teng et al., 2012). This architecture confers to the trachea also the ability to resist neck movements and pressure coming from the adjacent oesophagus. Moreover, being in direct contact with the outside world, the innermost part of the trachea is lined by a specialized and pseudostratified respiratory epithelium. This latter plays a central role within tracheal physiology, orchestrating one of the most intricated and sophisticated response mechanisms of the entire human body (Ganesan et al., 2013). Indeed, it not only acts as the primary physical barrier against environmental factors, continuously filtering, warming and humidifying the inhaled air, but it also perceives potential dangers and, in synergy with immune and nervous cells, puts in place the most appropriate response (Davis and Wypych, 2021). The complexity of the respiratory epithelium is reflected by the great heterogeneity encountered at the cellular level, in which each cell type plays a specific role within epithelial physiology (Goldfarbmuren et al., 2020). Thus, every alteration in cell content or selective damage to one or more cell types may contribute to the development of respiratory disorders (Davis and Wypych, 2021). Finally, the trachea is nourished by a multitude of tiny capillaries that branch mainly from the inferior thyroid artery and provide the blood supply to the pseudostratified epithelium and the surrounding tissues (Salassa et al., 1977). It is difficult to recreate or restore this intricated blood supply through anastomosis, therefore it appears as a major challenge in tracheal reconstructive attempts (Delaere et al., 2010).

**FIGURE 1:**
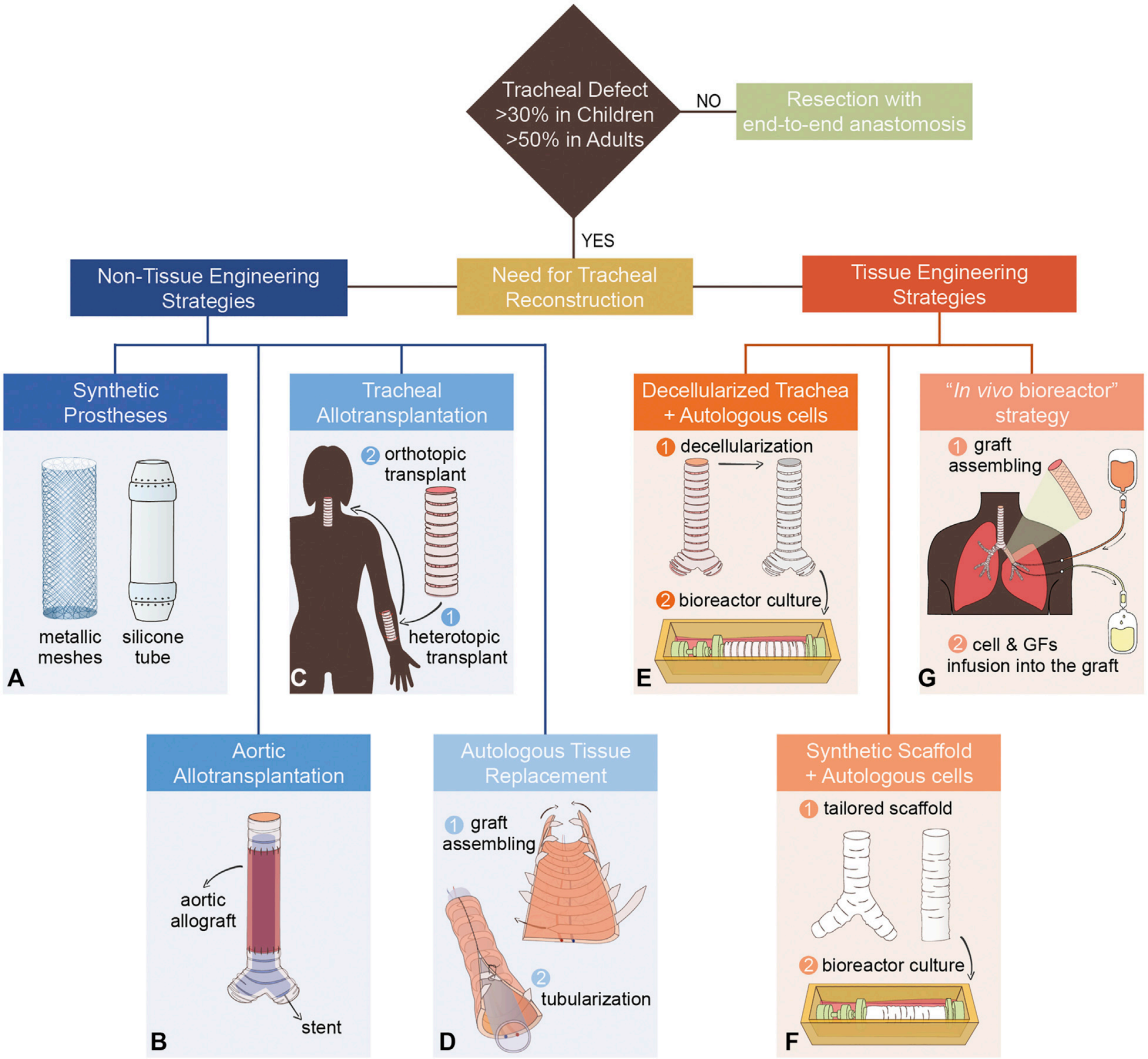
Examples of tracheal and main bronchi’s reconstructive strategies. **(A)** Synthetic prostheses: metallic meshes and silicone tube. **(B)** Aortic allotransplantation with a supporting stent. **(C)** Tracheal allotransplantation: two-step procedure with pre-vascularization in heterotopic position (forearm) followed by orthotopic transplantation. **(D)** Autologous tissue replacement: stripes of rib cartilage are inserted in a skin forearm free flap (graft assembling phase). Then, the graft is tubularized to reproduce the tracheal lumen. **(E)** An allogenic trachea is decellularized and then recellularized in a rotating bioreactor with autologous cells. **(F)** Synthetic tailored tracheal grafts seeded with autologous cells in a rotating bioreactor. **(G)** A graft composed of a nitinol stent inserted between two layers of porcine acellularized dermis matrix is seeded with autologous skin keratinocytes (graft assembling phase). Once transplanted, the graft is alternately perfused, through pumps and cannulas, with antibiotics, autologous cells, and growth factors (GFs).

This review is focused on the main clinical reconstructive strategies for replacing long-circumferential tracheal defects. Procedures relying only on external or internal splint/stent as a mechanical support to the weakened tissue and patch-based reconstructive approaches were not included. According to their manufacturing process, the revised approaches were classified in two macro-categories, namely non-tissue engineering (non-TE) and tissue engineering (TE) attempts (Figure 1). Indeed, the former comprises the use of synthetic prostheses or allogenic/autologous tissues directly transplanted *in vivo*, while the latter relies on an *in vitro* step of scaffold manufacturing and/or cell expansion. The clinical outcomes have been analyzed for each strategy to point out the achievements and the weaknesses that still hinder this field. Moreover, non-exhaustive examples of the forefront TE attempts on animal models were described as promising perspectives for the clinical translation. Thanks to this analysis, we identified some critical aspects that must be considered and implemented in designing new tracheal substitutes, thus paving the way towards safer and more effective solutions for treating patients with long-circumferential tracheal defects.

## Non-Tissue Engineering Approaches for Tracheal Restoration

In the last century, a plethora of non-TE approaches has been proposed and applied in small series or even single patients to treat long-circumferential tracheal defects. Based on common methodologies, these attempts can be further clustered into three main categories: synthetic prostheses, allotransplantations, and autologous tissue reconstruction. Table 1 summarizes some of the main non-TE approaches tested so far and still considered as potential tracheal reconstruction strategies.

**TABLE 1:** Major tracheal and main bronchi clinical reconstructive strategies.

NON-TE APPROACHES							
Approach	Patient details (years, gender, pathology)	Material	Cells	Details	Follow-up	Results	Authors
Allotransplantation	Six patients (17–52 yrs, 5 males, 1 female), extensive mucoepidermoid carcinoma (*n* = 1), adenoid cystic carcinoma (*n* = 5)	Stent-supported aortic allograft	N/A	Fresh (*n* = 2) or cryopreserved (*n* = 4) aortic allografts were wrapped with well-vascularized flaps of the pectoral muscle (*n* = 6) and, in two patients, with an additional thymopericardial flap to promote revascularization. A silicon stent was also implanted	26–45 months	Complete tumour resection was achieved in 83% of patients. Three patients suffered from fistulas, while adequate vascularization was observed in all cases. At the end of the follow-up, four patients were disease-free and still alive	Wurtz et al. (2010)

	Twenty patients (24–79 yrs,7 females, 13 males), non-small cell lung cancer (*n* = 11), postintubation tracheal stenosis (n = 3), carcinoid tumour (*n* = 3), thyroid carcinoma (*n* = 2) rhabdomyosarcoma (*n* = 1)	Stent-supported cryopreserved aortic allograft	N/A	A gender-mismatched –80°C cryopreserved aortic allograft was employed for airway reconstruction. A custom-made nitinol stent was inserted into the allograft to prevent airway collapse. No immunosuppressive therapy was required. 7 of the 20 patients were not eligible for transplantation	3–47 months	The overall mortality rate at 3 months was 5%. After a median follow-up of 47 months, 10 of the 13 transplanted patients were alive, with 8 of them showing normal breathing, regeneration of epithelium and *de novo* cartilage within aortic matrix	Martinod et al. (2018)

	1 patient (50 yrs, male), multiple tracheal stenosis and tracheomalacia due to intubation after SARS-CoV-2 infection	Stent-supported cryopreserved aortic allograft	N/A	A non-matched cryopreserved aortic allograft was anastomosed to the cricoid and carina, while a silicon stent was inserted to ensure patency. Both anastomoses were finally carefully covered by omental tissue	2 months	Two months postoperatively, the patient was able to autonomously clear secretions. Neither immunosuppression therapy nor routine bronchoscopy were required	Menna et al. (2021)

	1 patient (24 yrs, female), tracheal stenosis due to idiopathic fibrosing mediastinitis	Tracheal Allotransplantation	N/A	The allograft was wrapped within the omentum. Immunosuppressive therapy was required	12 months	Signs of rejection and necrosis were detected from day 10. A linear silicon endoprosthesis was required to face stenosis. At 1 year of follow-up, the patient was alive and with reduced signs of rejection	Levashov et al. (1993)

	6 patients (17–64 yrs, 3 males, 3 females), long-segment tracheal stenosis (*n* = 5), chondrosarcoma (*n* = 1)	Tracheal Allotransplantation	N/A	Allogenic tracheas were implanted in the forearm to improve vascularization and, in three patients, were repopulated with a patch of buccal mucosa	6–12 months	In three patients, tracheal necrosis and poor vascularization led to a partial loss of the allotransplant. The two patients that received oral mucosal cells and wrapping in the forearm fascia showed normal airways and no adverse events.	Delaere et al. (2010), Delaere et al. (2012)

	1 patient (43 yrs, female), adenoid cystic carcinoma	Tracheal Allotransplantation	N/A	After mechanical decellularization, the donor trachea was revascularized in the forearm of the patient. Seven weeks later, the vascularized allograft was orthotopically implanted	0.7 months	The patient was extubated on day 12. At day 22, a haemorrhage arised from the neotrachea in the mediastinum led to the patient’s dead	Iyer et al. (2020)
	1 patient (56 yrs, female), long-segment cricotracheal stenosis and tracheomalacia	Tracheal Allotransplantation	N/A	In this first single-stage human vascularized long-segment tracheal transplantation, the VCA was placed into the recipient bed. A microscope was used to perform the microvascular anastomoses. Triple immunosuppression was administered	6 months	The restoration of the blood supply was successfully obtained through microvasculature anastomoses. Imaging and bronchoscopic biopsies demonstrate graft vascularization and viable epithelial lining. Six months after transplantation, the patient was able to breathe without the need for tracheostomy or stent	Genden et al. (2021)
Autologous Replacement	1 patient (68 yrs, male), tracheal squamous cell carcinoma	Stent-supported aortic autograft	N/A	A 7 cm abdominal aorta autograft was harvested and replaced with a Dracon graft. A silicon Dumon stent was placed into the aortic graft to avoid aortic wall injury	6 months	An acute respiratory distress syndrome due to granulation required the application of an additional tracheal stent. The patient died at 6 months from septic shock after being treated for pneumonia and a controlateral pneumothorax	Azorin et al. (2006)

	16 patients (37–68 yrs, 7 males,9 females), adenoid cystic carcinoma (*n* = 9), squamous cell carcinoma (*n* = 3), tracheo-oesophageal fistulae (*n* = 2), thyroid cancer with tracheal invasion (*n* = 1), tracheal ischaemic stenosis (*n* = 1)	Fasciocutaneous skin flap reinforced with strips of rib cartilage	N/A	Forearm fasciocutaneous flap vascularized by radial vessels and reinforced through rib cartilages interposed transversally in the subcutaneous tissue. Construct was sutured before implantation	0.8–132 months	Two deaths in the postoperative period due to lung infections and acute respiratory distress syndrome, two deaths for cancer recurrence. Long-term follow-up analysis for 15 patients showed a 65% survival rate at 5 years	Fabre et al. (2013), Fabre et al. (2015)

	5 patients (28–52 yrs, 3 males, 2 females), primary tracheal malignant tumour (*n* = 1), right main bronchial stenosis (*n* = 1), left main bronchus tumour (*n* = 2), adenoid cystic carcinoma (*n* = 1)	Pulmonary tissue flap lined with an elastic metallic stent	N/A	Autologous pulmonary tissue flap lined with an elastic metallic stent to treat extensive tracheal resection	14–84 months	Bronchoscopy after 1 and 2 years detected neither stenosis nor perforation. One patient died at 14 months from severe haemoptysis, while the remaining patients were still alive after 84 months	Zhang & Liu, (2015)

	1 patient (25 yrs, male), postventilation tracheal stenosis	Cutaneous chondromucosal forearm tubular flap	N/A	A 4.5 cm-segment was replaced with strips of costal cartilage sutured around a segment of silicon, previously subcutaneously implanted in the forearm and lined with oral mucosal grafts	6 months	Postoperative analysis at 2 and 6 months revealed normal tracheal calibre, absence of granulation tissue, and a well-vascularized internal mucosal lining	Olias et al. (2005)

	1 patient (38 yrs, male), medullary thyroid carcinoma	Composite skin/omental /oesophageal graft	N/A	Tracheal continuity was restored through a 9 × 6 cm chest wall skin flap sutured to the still viable distal, proximal tracheal stumps and to the lateral oesophageal margins	24 months	After 7 days, a bronchoscopy revealing initial graft stenosis led to the implantation of an Ultraflex stent. 24 months after the operation, the patient was doing well, with no signs of recurrence	Spaggiari et al. (2005)
	1 patient (43 yrs, female), adenoid cystic carcinoma	Forearm free flap tubed around a stent	N/A	A forearm free flap was harvested and wrapped around an Ultraflex stent before implantation in a 6 cm tracheal defect	16 months	Acceptable function of the neotrachea in the immediate postoperative period. Proximal stricture, sputum retention, and recurrent pneumonia emerged in the following months. Death for malignant hypercalcemia at 16 months	Beldholm et al. (2003)

	1 patient (63 yrs, female), papillary thyroid carcinoma	Forearm free flap with an external mesh support	N/A	The reconstruction of the tracheal defect was obtained through a graft composed of a radial forearm fasciocutaneous free flap combined with a Hemashield vascular graft and reinforced with a PolyMax resorbable mesh	6 months	At 6 months, the patient was symptom-free and has returned to normal activities, with bronchoscopy showing a patent airway	Yu et al. (2006)
**TE APPROACHES**							
**Approach**	**Patient details (years, gender, pathology)**	**Material**	**Cells**	**Details**	**Follow-up**	**Results**	**Authors**
Allogenic decellularized tissues	1 patient (30 yrs, female), end-stage bronchomalacia	Decellularized human donor trachea	Epithelial bronchial cells and BM-MSC derived chondrocytes	A donor trachea was decellularized and recellularized with pre-expanded autologous cells. The graft was then used to replace the recipient’s left main bronchus	60 months	Continuous reinterventions were needed to remove the different stents rejected by the patient’s body and the granulation tissue responsible for stent obstruction	Macchiarini et al. (2008), GonfiOtti et al. (2014)

	1 patient (15 yrs, female), severe congenital malformations (a single left lung and long-segment congenital tracheal stenosis)	Decellularized human donor trachea	Autologous BM-MSCs and epithelial cells from the inferior turbinate	A donor trachea was decellularized with GMP-compliant reagents and recellularized in a bioreactor with autologous cells pre-expanded in a licensed cell therapy facility	0.5 months	Despite promising early results, an acute tracheal obstruction of the posterior wall occurred 2 weeks post-transplantation and led to the young girl’s death	Elliott et al. (2017)

	1 patient (10 yrs, male), long-segment congenital tracheal stenosis and pulmonary sling	Decellularized human donor trachea	Autologous BM-MSCs and patches of autologous epithelium	A decellularized trachea was saturated with hematopoietic stem cells, and patches of tracheal epithelium were secured to the graft’s lumen via a bioresorbable stent. GFs were administrated as pharmacological support	48 months	Many postoperative interventions were necessary, mainly to clear secretions, granulation, and remove a malacic graft segment. Four years after the transplant, the child was in good health, proving this procedure as lifesaving	Elliott et al. (2012), Hamilton et al. (2015)

	1 patient (38 yrs,1 female), Hodgkin lymphoma	AlloDerm (allogenic decellularized human derma)	N/A	The allogenic decellularized human derma was sutured into a tube and transplanted into the defect. Two different muscle flaps were used to cover and repair the chest and the neck	48 months	Postoperatively, the migration of the graft required its repositioning. Then, nine bronchoscopies (among which two dilatations) were necessary. Four years later, the patient is disease-free and lives a normal life	Bolton et al. (2017)
Patient-tailored synthetic scaffolds	3 patients (22–37 yrs, 2 males, 1 female), mucoepidermoid carcinoma (*n* = 1), adenoid cystic carcinoma (*n* = 1), iatrogenic tracheal injury (*n* = 1) carcinoma;	Nanocomposite polymer POSS-PCU (*n* = 1), electrospun polyblend of PET/PU 70%/30% (*n* = 2), electrospun PET 100% (additional implant, *n* = 1)	Autologous BM-MNCs	Synthetic tracheal grafts were seeded in a rotating bioreactor with autologous BM-MNCs in combination with locally and systemically GFs (TGF-b3, G-CSF and epoetin)	3,5–55 months	All patients developed graft-related complications and died after multiple surgical interventions. The main problems encountered were anastomotic fistulae, obstructive granulation tissue, absence of graft vascularization and mucosal lining	Fux et al. (2020)

“*In vivo* bioreactor” strategy	1 patient (57 yrs, male), squamous lung cancer	Nitinol stent and two layers of porcine ADM	Autologous skin keratinocytes and TNCs	A nitinol stent was enveloped between two layers of ADM. Skin keratinocytes were seeded on the lumen of the TE substitute. Once transplanted, Ringer’s solution and TNCs were injected into the graft	13 months	Bronchoscopy revealed signs of revascularization and biodegradation of the ADM scaffold. After 4 months, a biopsy showed epithelial tissue lining the graft. The patient died of lung cancer relapse 13 months postoperatively	Tan et al. (2017)

The table summarizes some of the main non-tissue engineering (non-TE) and tissue engineering (TE) approaches tested so far and still considered as potential strategies for long tracheal defects repair. For each approach, the number and the type of patients treated, the duration of the follow-up and the clinical results observed were reported. yr, year-old; N/A, not applicable; BM-MSCs, bone marrow-derived mesenchymal stromal cells; GFs, growth factors; GMP, good manufacturing practice; POSS-PCU, polyhedral oligomeric silsesquioxane–poly(carbonate-urea)urethane; PET/PU, polyethylene terephthalate/polyurethane; BM-MNCs, bone marrow–derived mononuclear cells; TGFß-3, Transforming Growth Factor beta-3; G-CSF, Granulocyte-Colony Stimulating Factor; ADM, Acellular Dermal Matrix; TNCs, total nucleated cells; TE, tissue engineering; VCA, vascularized composite allotransplantation.

### Synthetic Prostheses

Historically, tracheal reconstructive approaches involving rigid polymer constructs, glass or metal prosthesis, or more flexible materials such as polymeric or metallic wire/meshes, and silicone, have always been unsuccessful in clinical practice (Greaney and Niklason, 2021). Indeed, rigid polymer constructs or prostheses, despite remaining patent, are challenging to be properly sutured and often shifted out. The complication led to airway obstruction with consequent pneumonia or even death (Abbott et al., 1932; Clagett et al., 1948; Longmire, 1948; Cotton et al., 1952; Blades and Beattie, 1986). Moreover, these inert materials do not support the regeneration of the mucosal tissue, essential for a functional tracheal restoration. Similarly, silicone tubes have been used to exploit their durability, low reactivity, and mechanical flexibility. However, the lack of integration with the surrounding tissue, granulation formation and haemorrhages due to graft mobilization strongly limited their clinical diffusion (Neville et al., 1976; Neville et al., 1990; Toomes et al., 1985; Schneider et al., 2001). Finally, polymeric or metallic wire/meshes have also been adopted as tracheal substitutes, displaying unsafe and ineffective outcomes (Greaney and Niklason, 2021). Indeed, besides the usual granulation tissue formation, scaffold migration with erosion of the surrounding vessels and oedema, these prostheses lack adequate mechanical properties, leading to airway narrowing and collapse (Clagett et al., 1948; Kramish and Morfit, 1963). Therefore, to date, prosthetic reconstruction is no longer considered a therapeutic solution for long-circumferential tracheal defects.

### Tracheal Replacement With Allogenic Tissues

This strategy relies on the use of human cadaveric allogeneic tissues, among which aortic and tracheal allografts have been widely used.

#### Aortic Allotransplantation

Aortic allografts have been employed for tracheal repair by different groups, especially in emergencies, due to their wide availability in a variety of sizes (Davidson et al., 2009; Martinod et al., 2017, Martinod et al., 2018; Menna et al., 2021; Wurtz et al., 2006, Wurtz et al., 2010). However, these replacements lack the adequate biomechanical properties of the trachea, as well as the presence of respiratory epithelium. Therefore, to preserve the airway patency, aortic allografts required stenting, often rejected by the patient’s body. Moreover, several frequently fatal surgical-related complications were observed, such as severe bilateral pneumonia, anastomotic dehiscence, fungal infection, spinal cord ischemia and even cardiac arrest (Davidson et al., 2009; Wurtz et al., 2006, Wurtz et al., 2010). Additionally, these reconstructive approaches do not consider the need for an effective blood supply reestablishment, and rely only on host cells migration for graft repopulation.

In 2017, a clinical trial (TRACHEOBRONCART, NCT01331863) evaluated cryopreserved, stented aortic graft for tracheal replacement in patients affected by end-stage airway diseases (Martinod et al., 2017). So far, in the 13 patients reported, Martinod and colleagues showed that the cryopreserved aortic graft promoted the regeneration of new tissue (Martinod et al., 2017, Martinod et al., 2018). However, some stent-related complications such as infection and granuloma were also observed, suggesting that further studies are needed to confirm if this technique is safely applicable for long-circumferential damages or should be restricted to patch repairs only. Recently, a stented cryopreserved aortic allograft has also been used for the first time to treat a post-COVID-19 patient presenting multiple tracheal stenoses, tracheomalacia and ossification (Menna et al., 2021).

#### Tracheal Allotransplantation

Tracheal segments derived from brain-dead donors have been frequently used as non-vascularized allografts to treat patients with extensive tracheal damages. In 1993, Levashov et al. described a one-stage procedure consisting of direct trachea transplantation from a just passed away donor. Ten days postoperation, despite immunosuppression, signs of rejection appeared, followed by graft stenosis, which required the implantation of a silicone stent (Levashov et al., 1993). The direct tracheal allotransplantations outcomes confirmed these results, coupled with graft’s ischemia and necrosis within 2 months post-implantation. Formalin-fixed tracheas have been allotransplanted to reduce tissue immunogenicity, increase its stiffness and be readily available in case of emergency. However, despite early success, the long-term follow up revealed graft stenosis and the need for stenting, reasons why this procedure was almost completely abandoned (Greaney and Niklason, 2021).

In 2008, after studies in animal models and a first human preliminary report in 1979 (Rose et al., 1979; Delaere et al., 1995; Delaere et al., 1996), Delaere’s group performed a two-step tracheal allotransplantation in a patient with a long history of tracheal stenosis (Delaere et al., 2010). In this procedure, the trachea obtained from a brain-dead individual was first heterotopically implanted into the recipient’s forearm to promote vascularization and then transplanted in its orthotopic position. Additionally, the respiratory mucosa of the donor trachea was replenished by a flap of oral mucosa from the recipient, creating a chimeric graft. The same treatment was applied on six patients leading to several complications, principally related to rejection after withdrawal of immunosuppressant and graft necrosis, which led to a partial loss of the allograft (Delaere et al., 2012; Delaere and Van Raemdonck, 2016). In conclusion, the reconstructed airways were insufficient to sustain respiration, and required tracheostomy in some cases. Recently, the same technology has been applied by Iyer and colleagues to treat a patient affected by tracheal adenoid cystic carcinoma. However, soon after the surgery, a strong haemorrhage arising from the “neotrachea” led to the patient’s death (Iyer et al., 2020).

These poor outcomes point out some significant limitations related to this technology, including 1) dependence on donor availability and related blood matching (Delaere et al., 2010), 2) long time requirement for heterotopic pre-vascularization of the graft (inapplicable in emergencies), 3) extended periods of immunosuppression to limit rejection of the graft, which exposes the subject to opportunistic infections and that is inapplicable in case of cancer patients, and 4) altered mucociliary clearance, colonization with abnormal flora and secondary morbidity in case of heterotopic use of skin/oral mucosa flap to replace the necrotic tracheal epithelium (Udelsman et al., 2018).

Only recently, Genden and colleagues, after preclinical *in vivo* studies on animal models (Genden et al., 2002; Genden et al., 2003), tried to overturn a longstanding dogma for which the tracheal microvascular anastomoses is not achievable (Genden et al., 2021). Specifically, Genden performed a single-stage tracheal vascular composite allotransplantation (VCA) involving the thyroid arteries and the muscular wall of the donor’s oesophagus that shares blood supply with the donor trachea. The restoration of the blood supply was successfully obtained through microvasculature anastomoses, and a well-perfused continuous airway respiratory epithelium was observed via endoscopy and histological examination. However, even if this approach addresses some of the problems faced in the two-step tracheal allotransplants, the need for immunosuppression still represents a limitation restricting this approach to non-oncologic patients only. Moreover, a longer follow-up and a larger cohort of patients are required to evaluate the safety and the efficacy of this procedure (Randhawa and Patterson, 2021).

### Tracheal Replacement With Autologous Tissues

Finally, a different approach involves multiple autologous tissues as a source for tracheal and main bronchi reconstruction, such as aortic autograft (Azorin et al., 2006), pulmonary tissue (Zhang and Liu, 2015), skin (Spaggiari et al., 2005), intercostal artery muscle flap (Bertheuil et al., 2021), forearm flaps and costal cartilage (Beldholm et al., 2003; Olias et al., 2005; Yu et al., 2006; Fabre et al., 2013; Mercier et al., 2018). The common goal of these reconstructive techniques is to generate a tubularized and well-perfused graft to replace the damaged trachea. In many cases, this was combined with a temporary or permanent stent to give structural stability to the transplant (Azorin et al., 2006; Fabre et al., 2013; Zhang and Liu, 2015). In 2013, after preclinical studies on large animal models (Fabre et al., 2009), Fabre and colleagues published the largest series of patients treated with autologous tissue for airway replacement purposes (Fabre et al., 2013, 2015). They used a single-step procedure to generate and transplant a tracheal substitute made by regular intervals of forearm free fasciocutaneous flaps and costal cartilages. Following this procedure, seven out of twelve patients developed acute respiratory distress syndrome, and two became tracheostomy dependent (Udelsman et al., 2018).

Although these innovative approaches allow avoiding immunosuppression, the variable clinical outcomes highlight several issues, such as 1) the need for temporary or permanent stents, 2) the absence of an integer epithelium, which is crucial for a physiologic mucociliary clearance, and 3) the risk of morbidities at the donor site following the withdrawal of autologous tissue.

## Tissue Engineering Approaches for Tracheobronchial Reconstruction

Tissue engineering (TE) approaches rely on the combination of cells with an appropriate scaffold for the treatment of significant tissue defects. Indeed, in this interdisciplinary field, principles of biomaterial engineering, genetics, cell biology and clinical science are combined to develop a biological graft to maintain, restore or improve tissues or whole organs (Shafiee and Atala, 2017; Vranckx and Hondt, 2017). This solution can simultaneously address different problems, such as donor shortage (often fatal for patients in critical status, struggling with long waiting lists), immunosuppression therapy and donor site morbidity. In fact, TE strategies take advantage of the body’s regenerative potential and, by *in vitro* expansion, allow to obtain enough cells to regenerate extensive body areas from a small biopsy (Gallico, 1985; Corradini et al., 2016). Thus, this field represents a promising strategy for expanding the current reconstructive armamentarium to treat severe unmet medical needs (Vranckx and Hondt, 2017).

Between 2008 and 2017, TE attempts for windpipe and main bronchi’s replacement have been tested in compassionate cases, and the trachea has been acclaimed by both the scientific community and mass media as the first tissue-engineered organ (Fountain, 2012). However, this field was impaired by a history of scientific and ethical misconduct (The Lancet, 2018; The Lancet Editors, 2018; Jungebluth et al., 2019), which has led to a general sense of mistrust concerning airway TE potential.

To date, three main strategies have been proposed in tracheal TE, which involves the use of allogenic decellularized human cadaveric donor tissue, synthetic patient-tailored scaffolds, or an “*in vivo* bioreactor” strategy. Table 1 summarizes some of the main experiences in this field.

### Allogenic Decellularized Tissues

Among the different scaffolds used for TE approaches, the extracellular matrix (ECM) obtained through decellularization of allogenic tissues has been used for tracheal replacement. An example of allogenic tissue devoided of the cellular component is the extracellular dermal matrix, recently used as non-vascularized graft for long-segmental tracheal replacement (Bolton et al., 2017). However, this non-resolutive strategy required postoperative refinement due to graft migration. Moreover, this attempt relies only on cell migration from the wound edges, insufficient in case of extensive defects. On the other hand, the ECM obtained from allogenic human cadaveric tracheas has been adopted either with cultured autologous cells or freshly harvested hematopoietic stem cells.

#### Allogenic Tracheal Extracellular Matrix Plus Cultured Cells

The first patient to receive a TE approach was a 30 year-old woman suffering from end-stage bronchomalacia (Macchiarini et al., 2008). In this compassionate case, a human donor trachea was decellularized with an extensive protocol to obtain a suitable scaffold to be colonized with autologous cells. Specifically, epithelial cells isolated from a bronchus’ mucosal biopsy were cultured under serum-free conditions, while mesenchymal stem cells from bone marrow aspirate were expanded and induced to differentiate in chondrocytes (Macchiarini et al., 2008). Through a perfusion system, a bioreactor with two separate accesses was used to seed epithelial cells onto the internal surface of the decellularized trachea. At the same time, the chondrocytes were injected into the external surface of the matrix. Finally, after surgical removal of the damaged tissue, the avascular graft was shaped and sutured to the remaining native tissue (Macchiarini et al., 2008). In 2014, Gonfiotti et al. reported the patient’s 5-year follow-up, describing this TE approach as safe and promising.

However, the postoperative course was characterized by many complications. The development of granulation tissue and cicatricial scar led to the implantation of several endoluminal stents to maintain the airway patent. Continuous reinterventions were necessary to remove the different stents rejected by the patient’s body and the granulation tissue responsible for stent obstruction (Gonfiotti et al., 2014). Such graft-related postoperative complications strongly affected the patient’s quality of life (Molins, 2019).

Later on, Elliott and colleagues adopted a similar technique to treat a 15 year-old girl born with severe congenital malformations (a single left lung and long-segment congenital tracheal stenosis) after other failed reconstructive approaches (Elliott et al., 2017). In this study, every step of the reconstructive procedure was designed to fulfil good manufacturing practice (GMP) standards. Specifically, a donor trachea was decellularized with GMP-compliant reagents. Meanwhile, autologous bone marrow cells and epithelial cells from the inferior turbinate were expanded in a licensed cell therapy facility and then seeded onto the decellularized matrix in a bioreactor (Elliott et al., 2017). Despite promising early results, an acute tracheal obstruction of the posterior wall occurred 2 weeks post-transplantation and led to the young girl’s death. Given this negative outcome, the authors recommended using stents during the first few months postoperatively. Moreover, they highlighted the difficulties in translating a TE reconstructive approach from the preclinical setting to the clinic, even because the *in vivo* models cannot mimic complex clinical scenarios (Elliott et al., 2017).

#### Allogenic Tracheal Extracellular Matrix Plus Freshly Harvested Cells

To reduce the time needed for producing a TE tracheal substitute, a decellularized trachea was saturated with freshly harvested hematopoietic stem cells. Besides, patches of tracheal epithelium were placed as free grafts within its lumen and secured via a bioresorbable stent. This TE attempt aimed to recreate an *in vivo* microenvironment, recapitulating some of the key stimuli that lead to the physiological post-injury repair. In this respect, several growth factors were systemically administrated both in the preoperative and postoperative periods and locally injected into the avascular TE construct during the implantation. This pharmacological support aimed to mobilize haemopoietic stem cells and endothelial progenitors, improve mesenchymal stromal cells (MSCs) recruitment, induce chondrocyte differentiation, and increase angiogenesis (Elliott et al., 2012). The 4-year follow-up of a 10 year-old child treated with this approach declared the boy’s good health, assessing this procedure as lifesaving (Hamilton et al., 2015). Despite this, many reinterventions (more than 25) were needed, especially during the first year of follow-up, to clear secretion, remove granulation tissue and replace the resorbable stent. Difficulties in the re-epithelialization of the decellularized tracheal matrix have been observed. Indeed, the histological analysis of a biopsy from the TE graft at 1 month post-intervention revealed granulation tissue only. Instead, a biopsy of the proximal transplant collected at 42 months, showed an epithelial layer with a mix of squamous and respiratory epithelium and only a few ciliated cells. Probably, the graft vascularization was insufficient to allow the epithelialization of the decellularized matrix from the free graft’s patches of tracheal mucosa, and the poor epithelialization occurred from the migration of cells from the wound edges. Nonetheless, this mechanism can cover only a few millimetres of the graft, while the central tissue is left as uncovered granular tissue (Delaere et al., 2014).

### Patient-Tailored Synthetic Scaffolds

Since 2011, synthetic tailored scaffolds repopulated by autologous bone marrow cells and supported by growth factors have been proposed to revolutionize regenerative medicine. Those constructs were made of nanocomposite polymer POSS-PCU (polyhedral oligomeric silsesquioxane–poly (carbonate-urea) urethane) or electrospun polyblend PET/PU (polyethylene terephthalate (PET) and polyurethane (PU)) (Fux et al., 2020) and were described as able to integrate within the recipient, generating living and functional grafts covered by epithelium (Del Gaudio et al., 2014; Jungebluth et al., 2011, Jungebluth et al., 2013). However, these papers were retracted for scientific misconduct in the following years. Only recently, Fux and colleagues have unequivocally stated the inadequacy of those constructs for TE purposes. In this retrospective study, the authors presented the first long-term follow-up of three patients, who in total received four synthetic tracheal grafts recellularized with bone marrow–derived mononuclear cells (BM-MNCs). During the postoperative period, all patients developed graft-related debilitating complications. Follow-up analysis showed the formation of anastomotic fistulae and obstructive granulation tissue as well as the absence of vascularization, epithelial lining, or integration within the surrounding tissue. All patients died after multiple surgical reinterventions, revealing the failure of TE synthetic tracheal substitutes as living functional grafts (Fux et al., 2020). Indeed, the “bioengineered” constructs behaved only like an inert scaffold, similarly to synthetic tracheal prostheses (Delaere et al., 2019).

### “*In Vivo* Bioreactor” Strategy

To face the challenges related to the delayed revascularization process and infections, Tan et al. proposed the use of the recipient body as a bioreactor for the TE substitute (Tan et al., 2017). This approach aims to combine the commonly separated *in vitro* 3D cell-scaffold culture with the *in vivo* regenerative process. With this purpose, a nitinol stent - providing lateral rigidity and longitudinal flexibility to the TE construct - was surrounded by two layers of a biodegradable porcine acellularized dermis matrix (ADM). One hour before transplant, the luminal side of the scaffold was seeded with epidermal keratinocytes obtained from the digestion of a skin flap. Two catheters were inserted among the two ADM layers and associated with peristaltic pumps during the implantation. Through this cannulation system, the avascular TE graft was perfused for 1 month with Ringer’s gentamicin fluid to prevent infection and to keep the epithelial cells alive before final revascularization. Moreover, this system allowed the secondary infusion of total nucleated cells (TNCs) and growth factors to stimulate graft regeneration directly into the transplanted TE construct. Unfortunately, the authors reported the patient’s death 13 months postoperatively. Consequently a longer follow-up is unavailable (Tan et al., 2017). Major doubts remain regarding the durability and functionality of this bioengineered construct. In fact, the gradual biodegradation of the ADM scaffold, observed during the postoperative bronchoscopies, and the use of epidermal keratinocytes instead of airway ciliated epithelial cells could be responsible for long-term severe graft-related complications.

## Frontiers of Tracheal Substitutes: Lessons From Preclinical Animal Studies

Failures and controversies raised by the clinical application of several tracheal substitutes have led to a renewed interest in preclinical studies based on animal models. According to Niermeyer et al., 73% of articles focused on tracheal reconstruction and published between 2015 and 2020 involved an *in vivo* preclinical model. This reveals the need for a more in-depth preliminary analysis of the tracheal substitutes before embarking on new clinical applications (Niermeyer et al., 2020).

This paragraph focuses on the latest *in vivo* preclinical TE attempts, since they stand as one of the most promising approaches to face hurdles that still hinder this field. Indeed, articles concerning tissue-engineered tracheal grafts (TETGs) aim to improve graft integration within the recipient, mimick complex native trachea biomechanics and prevent host adverse responses (Greaney & Niklason, 2021). To give an idea of the possible future clinical applications, a few non-exhaustive examples belonging to the different categories of the current TE approaches are listed below and summarized in Table 2.

**TABLE 2:** Preclinical animal studies.

Approach	Animal model, samples size	Scaffold material	Cells	Graft length	Follow-up	Outcomes	Lessons	Authors
Biosynthetic scaffolds	Sheep, *n* = 8	PET/PU reinforced with clinical-grade PC rings	autologous BM-MNC	5 cm	3–16 weeks	Graft stenosis, infections, mechanical failures, and lack of epithelialization were observed in all animals	The lack of epithelization and inappropriate blood supply causes a pro-inflammatory response leading to stenosis and graft failure	Pepper et al. (2019)

	Mice, *n* = 25	nonresorbable PET/PU and resorbable PGA/PLCL polymers	N/A	0.5 cm	1–8 weeks	Stenosis manifested in both groups, leading to premature death with respect to the study endpoint. Lack of respiratory epithelium in the mid-graft region	Graft stenosis was due to new tissue overgrowth in nonresorbable scaffolds and to malacia in resorbable scaffolds	Dharmadhikari et al. (2019)

	Rabbit, *n* = 18	PCL bellows scaffold reinforced with silicon rings and collagen	human turbinate mesenchymal stromal cell (hTMSC) sheets	1.3 cm	4 weeks	The graft lumen was covered by adjacent respiratory epithelium. Mild mucosal granulation was observed	PCL bellows graft could be promising for tracheal replacement, however acute rejection signs were observed	Lee et al. (2018)

	Porcine, *n* = 7	PCL and bovine decellularized ECM	N/A	4 cm	4–12 weeks	Graft lumen was covered by ciliated epithelium along with metaplastic squamous epithelium. Mild granulation tissue was revealed	Possible explanations of the granulation tissue formation could be rapid resorption of acellular scaffold and the absence of an epithelium at the time of implantation	Rehmani et al. (2017)

	Rabbit, *n* = 20	PCL	chondrocytes from rabbit auricle	1.6 cm	2–10 weeks	All animals died for granulation formation, pneumonia, infections, and stenosis. Absence of epithelium on the scaffold lumen surface	The graft had good cartilaginous properties. However, the lack of an epithelial layer and host inflammatory reactions caused stenosis and granulation formation	Gao et al. (2017)

	Rabbit, *n* = 11, Monkey, *n* = 3	PGA and nitinol stent	smooth muscle cells removed through decellularization before transplantation	0.8 cm length in rabbits; 1.8 cm length in monkeys	1–8 weeks	The implanted graft was well integrated, with no signs of collapse or infections. A ciliated epithelium covered its lumen. However, several strictures were observed at different time points	The acellular tissue-stent graft showed good biomechanical properties and proved to be pro-angiogenic *in vivo*, but still affected by stenosis related to delayed epithelialization	Zhao et al. (2016)

	Dog, n = 5	Collagen-coated nitinol frame	N/A	2 cm	4–96 weeks	4/5 dogs survived 18–24 months without signs of tracheal stenosis. Angiogenesis was observed in 3 months, and a good biocompatibility was confirmed	This artificial graft reproduced the physical properties of the native trachea. Regeneration of a ciliated epithelium was revealed, but as a monolayer rather than a pseudostratified columnar epithelium	Sakaguchi et al. (2018)

	Rabbit, *n* = 16	3D printed PLLA scaffold	autologous chondrocytes from rabbit auricle	1.5 cm	8 weeks	Animals in the control group (*n* = 8) whose scaffold was not pre-vascularized died for chronic tracheal stenosis within 1 month. Instead, 6/8 animals whose scaffold was *in vivo* pre-vascularized for 2 weeks survived at 2 months, showing an open lumen, with little occurred granulation tissue	Pre-vascularization process supports the regeneration of cartilage tissue and seeded cells’ survival, allowing to obtain an epithelialized lumen within the engineered trachea	Gao et al. (2019)
	Rabbit, *n* = 23	Electrospun PCL nanofibers covered by 3D printed PCL microfibers	hBECs, iPSC-derived MSC or iPSC-derived chondrocytes	1.5 cm	4 weeks	At the study endpoint of 4 weeks, the engineered trachea appeared covered by epithelium without severe granulation in both groups receiving scaffolds with hBECs and IPSC either derived from MSC or chondrocytes. Moreover, the group receiving IPSC-derived MSC showed fully differentiated epithelium with cilia formation	iPSC-MSCs may have a possible beneficial role in promoting the re-epithelialization process through paracrine mediators	Kim et al. (2020)

	Rat, *n* = 9	Custom-made casting molds of rat fibroblasts and collagen hydrogels	Rat fibroblasts and osteogenically-induced MSC	0.5 cm	24–48 h	3/9 rats died before 48 h, showing some strictures in anastomotic regions, 6 rats died during the operation because they could not be weaned from the respirator because of the impaired bioartificial trachea	The lack of epithelial lining on the lumen of the trachea is a great limitation. Thus, epithelial cells should also be considered within this approach	Naito et al. (2011)

Decellularized scaffolds	Porcine, *n* = 20	Porcine Decellularized trachea	Autologous MSCs-derived chondrocyte and bronchial epithelial cells	6 cm	1.5–8.5 weeks	Only the animals in which the decellularized matrix was seeded with both epithelial and chondrocytes were healthy and without signs of stenosis, infections, and rejection	Matrix seeding with both epithelial and mesenchymal stem cell–derived chondrocytes is required to obtain a functional graft	Go et al. (2010)

	Rabbit, *n* = 16	Decellularized rabbit trachea compared to preserved allograft and synthetic scaffold (POSS-PCU)	N/A	2 cm	1,5–4 weeks	Due to respiratory distress, all animals were early terminated. Graft malacia was observed as well as the absence of epithelization	Stenosis were observed in all groups, suggesting the necessity to evaluate seeded scaffold for tracheal replacement	Maughan et al. (2017)

Scaffold-free constructs	Rat, *n* = 9	Scaffold-free construct supported by a silicone stent	rat chondrocytes, endothelial cells and MSCs	0.48 cm	∼ 3 weeks	Vasculogenesis and chondrogenesis were observed. However, the lack of a luminal epithelium and the presence of a stent provoked granulation formation	The graft was sufficiently strong to be transplanted but required stent support to prevent graft collapse	Taniguchi et al. (2018)

	Rat, *n* = 3	Scaffold-free construct supported by a silicone stent	human chondrocytes, MSCs, fibroblasts, and human umbilical vein endothelial cells (HUVECs)	0.38 cm	5 weeks	The presence of epithelial cells from the native trachea and capillary-like structures were confirmed. The strength of the graft was lower than the native trachea	This technique could produce grafts made by human cells only. However, it still presents some limits such as a prolonged culture time to obtain a sufficient number of cells and the need for a stent	Machino et al. (2019)

The table summarizes some of the most recent *in vivo* preclinical strategies adopted to improve tracheal and main bronchi reconstruction. For each attempt, the animal model, the number of animals treated, the follow-up duration, the outcomes and the lessons learned have been reported. PET/PU, polyethylene terephthalate/polyurethane; PC, polycarbonate; PGA/PLCL, polyglycolic acid/poly(l-lactide-co-ε-caprolactone); PCL, polycaprolactone; ECM, extracellular matrix; BM-MNC, bone marrow mononuclear cells; POSS-PCU, polyhedral oligomeric silsesquioxane-poly(carbonate-urea)urethane; MSCs, mesenchymal stromal cells; PLLA, poly (L-lactic acid); hBECs, human bronchial epithelial cells; iPSC, induced pluripotent stem cells.

Concerning the use of synthetic material, several methodologies have been investigated to produce tubular scaffold suitable for tracheal replacement. In 2011, a casting-based manufacturing process was used to create a tracheal-shaped substitute starting from mixtures of rat fibroblast/MSCs and collagen hydrogels. The stiffness of the resulting bioartificial trachea was compared to the one of the native trachea showing no differences. However, when the former was transplanted in a rat model, only three of the nine treated animals survived the implantation, dying within 48 h after surgery. The author suggests that these results could be related to the absence of epithelial cells on the inner layer of the transplanted bioartificial trachea (Naito et al., 2011).

Some years later, Pepper et al. employed a polyethylene terephthalate (PET) and polyurethane (PU) scaffold combined with polycarbonate rings. This structure was seeded with BM-MNCs and transplanted in eight sheep to replace a 5 cm long tracheal defect. Despite promising mechanical tests, all animals showed graft stenosis associated with granulation tissue. Overall, this attempt remarked the centrality of the epithelialization and neovascularization, in the absence of which the outcomes are poor (Pepper et al., 2019).

Using a mouse model, another group compared synthetic non-resorbable PET/PU *vs.* resorbable poly(l-lactide-co-ε-caprolactone)/Polyglycolic acid (PLCL/PGA) scaffolds. Even in this study, graft’s stenosis was revealed in both conditions, with no signs of respiratory epithelization in the central part of the grafts (Dharmadhikari et al., 2019).

Several groups also investigated synthetic scaffolds based on polycaprolactone (PCL), given its easy moldability through 3D printers (Gao et al., 2017; Rehmani et al., 2017). To mimic the tracheal structure and mechanical profile, Lee and colleagues used a bellows-designed PCL scaffold reinforced with collagen, silicon rings and seeded with human turbinate mesenchymal stromal cells (hTMSC) sheets (Lee et al., 2018). After implantation in rabbits, the PCL was successfully incorporated within the adjacent tissue and lined by airway epithelium. However, respiratory distress and mild granulation process were observed in all animals. Additionally, the higher levels of interleukin-2 and interferon gamma detected in treated animals, compared to the baseline values, suggested a possible acute rejection (Lee et al., 2018). Two years later, another group used both electrospinning and 3D printing techniques to generate a PCL synthetic scaffold for tracheal reconstruction in a rabbit model. In this study, human bronchial epithelial cells (hBECs) were used to populate the inner layer of the PCL scaffold, while the outer layer was repopulated by either induced pluripotent stem cells-derived mesenchymal stem cells (iPSC-MSCs) or induced pluripotent stem cells-derived chondrocytes (iPSC-Chds). A regenerated epithelium was observed in both conditions at the study endpoint (4 weeks). However, in the iPSC-MSCs group, the epithelium was fully specialized and better organized than in the iPSC-Chds group, suggesting a paracrine effect of iPSC-MSCs in promoting the re-epithelialization process (Kim et al., 2020). To overcome the aforementioned problems related to synthetic tracheal TE grafts, other groups tried to reduce the immunogenicity of the grafts, increasing their survival prospects through pre-implantation vascularization strategies (Soriano et al., 2021). For example, Zhao et al. seeded smooth muscle cells onto a PGA-nitinol stent scaffold to allow the deposition of a collagenous matrix and increase the scaffold angiogenetic properties. The resulting construct was then decellularized, preserving the new extracellular matrix, and implanted in rabbits and nonhuman primates. No graft collapse was observed, while a columnar respiratory epithelium lined the construct lumen. However, mid-grafts stenosis was present (Zhao et al., 2016). In the preclinical study performed by Sakaguchi and colleagues, 80% of dogs transplanted with a pre-vascularized collagen-coated nitinol scaffold survived for more than 18 months. No signs of stenosis were reported, while a monolayer of ciliated cells covered the graft’s lumen. These promising results may be due to the pre-vascularization obtained through the graft’s heterotopic implantation into the omentum (Sakaguchi et al., 2018). Similar beneficial effects of the pre-vascularization process were observed in a rabbit preclinical study where a 3D printed PLLA (poly L-lactic acid) scaffold seeded with autologous chondrocytes was used to reconstruct a 1.5 cm long tracheal segment. Indeed, the engineered pre-vascularized trachea appeared integrated within the adjacent tissues and covered by respiratory epithelium. Besides, no signs of stenosis were observed with only sporadic granulation tissue formation (Gao et al., 2019).

Besides synthetic scaffolds, decellularized grafts have been employed in preclinical settings. However, when used without the cellular component, they behaved similarly to the polyhedral oligomeric silsesquioxane–poly (carbonate-urea)urethane (POSS-PCU) synthetic scaffolds, mainly developing stenosis (Maughan et al., 2017). Otherwise, when recellularized with epithelial cells and MSC-derived chondrocytes, they become functional and well-tolerated, without signs of rejection or airway collapse (Go et al., 2010). These results remark the need for the cellular component to promote graft re-epithelialization and structural stability essential for preventing host inflammatory response and graft failure.

Finally, new frontiers may arise from scaffold-free constructs. Indeed, through a novel Bio-3D printing system, many groups developed artificial tracheas assembling spheroids composed of various cell types, such as chondrocytes, MSCs and endothelial cells (Taniguchi et al., 2018; Machino et al., 2019). In these studies, to obtain a structure that recapitulates the tracheal shape, the multicellular spheroids were first placed into needle arrays and then underwent a maturation phase inside a bioreactor. Finally, the artificial scaffold-free constructs were implanted in rat models to evaluate the graft performance. At the end of the studies, these artificial tracheas showed signs of chondrogenesis, vasculogenesis, and an epithelium lining the lumen. However, the need for a permanent stent to avoid graft collapse and the presence of granulation tissue represent some limitations of this innovative technique (Taniguchi et al., 2018; Machino et al., 2019).

## Discussion

Tracheal and main bronchi dysfunctions represent an unmet and growing medical need, especially in the case of wide circumferential structural alterations where available surgical strategies are ineffective or inapplicable. The long-term clinical outcomes of reconstructive approaches tested so far clearly point out the difficulty of restoring a functional trachea (Grillo, 2002; Soriano et al., 2021).

Since the first challenge of any tracheal replacement’s attempt is to reproduce or mimic the native tracheal structural properties, a suitable tracheal substitute must fulfil specific biomechanical requirements. Indeed, it has to be airtight and possess longitudinal flexibility and lateral rigidity to withstand forces arising from respiration, coughing, neck movements and pressure created by the adjacent oesophagus (Grillo, 2002; Boazak and Auguste, 2018). Former approaches not fulfilling these requirements resulted in airway collapse, strictures, graft migration, or haemorrhages which, altogether, stand as the primary cause (68%) of graft-related mortality (Greaney and Niklason, 2021). Consequently, a preliminary assessment of the tracheal substitute’s suitability needs to be performed to reduce avoidable life-threatening adverse events on the patients. Indeed, several *in vivo* preclinical studies have been carried out to evaluate biochemical properties of decellularized (Zhao et al., 2016), synthetic (Dharmadhikari et al., 2019) or scaffold-free constructs (Machino et al., 2019). However, since the first requirement for an adequate tracheal substitute is to mimic the tracheal’s native biomechanical properties, the definition of standard approaches and biomechanical tests would be extremely helpful to obtain comparable results among different studies and further improve this field (Martínez-Hernández et al., 2021).

Another limitation for a successful reconstructive approach is the lack of studies on the biocompatibility between the tracheal substitute and the host. As previously described, this problem has been reported to trigger acute rejection, granulation tissue formation and graft necrosis, especially in the case of synthetic prostheses (Neville et al., 1976, Neville et al., 1990; Toomes et al., 1985), bioprosthetic substitutes (Hoffman et al., 2001) and tracheal allograft (Levashov et al., 1993; Delaere et al., 2010; Delaere et al., 2012). Before implantation, specific biocompatibility studies should be performed to ensure that the tracheal substitute is made of safe, non-immunogenic material, well-tolerated by the recipient and able to integrate within the body. In order to avoid the aforementioned rejection problem, particularly marked in the case of allograft, also autologous tissues have been clinically applied (Olias et al., 2005; Spaggiari et al., 2005; Fabre et al., 2013, Fabre et al., 2015; Zhang and Liu, 2015; Mercier et al., 2018). However, even this procedure presented some problems, such as donor-site morbidity (Udelsman et al., 2018). In this scenario, TE represents a unique opportunity to rebuild extensive body surfaces, combining cells extracted from a small autologous biopsy with an appropriate scaffold (Shafiee and Atala, 2017; Vranckx and Hondt, 2017).

Regardless of the reconstructive approach, one of the common hurdles in tracheal replacement is graft epithelialization. Indeed, airway functions are critically dependent on the respiratory epithelium, and its absence triggers several complications, including mucous stagnation, infections, graft stenosis, granulation tissue formation, chondromalacia and fibrogenic reaction (Heijink et al., 2014; Paternoster and Vranckx, 2021). Therefore, any tracheal replacement attempt should be aimed at restoring a functional and well specialized respiratory epithelium (Davis and Wypych, 2021). Several aspects must be considered to ensure the presence of a continuous, self-renewing and specialized respiratory epithelium lining the lumen of the tracheal substitute. First, due to the respiratory tissue’s high complexity and cellular heterogeneity, epithelial cells or sheets derived from other autologous districts (i.e., skin or oral mucosa) cannot be used for airway functional recovery (Tan et al., 2017; Udelsman et al., 2018). Second, spontaneous post-surgery reepithelization of the tracheal substitute cannot be taken for granted. In clinical and preclinical studies, several groups relied on the migration of epithelial cells from the recipient’s wound edges (Martinod et al., 2017, Martinod et al., 2018; Rehmani et al., 2017), with some successful epithelialization only on short-sized scaffolds (Zhao et al., 2016; Lee et al., 2018; Sakaguchi et al., 2018). Indeed, local epithelial cells can only cover a few millimeteres of the graft, while the central part of the tracheal substitute is often left uncovered, leading to the aforementioned adverse consequences of an absent epithelium (Delaere and Van Raemdonck, 2019). Therefore, a precise and rational reepithelization strategy is mandatory, especially for tracheal substitutes of larger dimensions. On this note, in TE approaches, the culture conditions used for the *in vitro* expansion phase must preserve the proliferative and differentiative potential of the airway epithelial stem cells, preventing their exhaustion. Indeed, the experience gained from regenerative approaches of other human epithelial tissues clearly pointed out that a specific number of stem cells is strictly required to allow the permanent engraftment, renewal, and restoration of the epithelium (Pellegrini et al., 2011; Maurizi et al., 2021). In order to carefully monitor these aspects and have a clear overview of all the process variables, extensive cellular characterization must be performed. This comes through the identification of population-specific molecular markers to be adopted as quality controls during each step of the reconstructive procedure.

To date, there is a huge gap between basic-research knowledge on airway epithelial cells’ biology and the application of these insights to TE translational approaches. Indeed, despite the huge work carried out to understand the airway epithelial cells physiology and heterogeneity (Garcıá et al., 2019; Basil et al., 2020; Goldfarbmuren et al., 2020; Zaragosi et al., 2020; Davis and Wypych, 2021), the few groups that clinically employed airway epithelial cells for tracheal TE approaches did not exploit this knowledge, reporting only limited or inadequate cellular characterization (Macchiarini et al., 2008; Elliott et al., 2017).

In order to bridge this gap, preclinical studies—especially those based on *in vitro* models—can be extremely useful to precisely evaluate all the interactions among the different components of the bioengineered construct. Indeed, once in contact with the scaffold, colonizing cells should retain their ability to proliferate and differentiate into their respective specialized cell types, a crucial aspect for re-establishing the system’s physiology. Moreover, in the case of TE approaches encompassing cells derived from different tissues (i.e., epithelial cells, chondrocytes, endothelial cells, neural cells etc.), their mutual interaction must be carefully studied to exclude possible acute or chronic cytopathic effects.

Another common issue with all the tracheal reconstructive approaches is the vascularization of the tracheal substitute. Till now, this aspect has deeply hampered the outcomes of treated patients in several clinical studies (Delaere et al., 2012; Delaere et al., 2019). Indeed, efficient and rapid restoration of the blood supply is mandatory to sustain graft survival, allow its integration within the surrounding tissue, avoid necrosis or contamination, and support new cartilage and epithelium regeneration. Some approaches tried to overcome this matter by wrapping tracheal substitutes in a highly vascularized tissue, frequently transposed omentum (Levashov et al., 1993; Elliott et al., 2012; Menna et al., 2021). Although these efforts have been initially interpreted as successful, long-term follow-ups demonstrated that, in most cases, this strategy only temporarily delays the inevitable consequences of wound breakdown at the anastomoses (Delaere and Van Raemdonck, 2014). Alternatively, tracheal substitutes can be *in vivo* vascularized through heterotopic implantation into the recipient’s forearm, followed by orthotopic repositioning. Still, such strategies’ long-time requirements and invasiveness have strongly limited their application. Once more, a possible solution may arise from *in vitro* or *in vivo* preclinical studies investigating the formation of a vascular network through interaction with endothelial cells or from stimulating the revascularization process through the delivery of pro-angiogenic factors (Lovett et al., 2009).

Alongside vascularization, restoring a functional innervation system within the tracheal substitute should be considered. Indeed, the airway epithelium works conjointly with the immune and nervous systems to guarantee respiratory homeostasis (Davis and Wypych, 2021). So far, this aspect has been neglected, as no significant clinical studies have encompassed the inclusion of this component. Thus, it remains a crucial issue to be addressed in the future.

Finally, another critical point to be mentioned regards the legislative side. Since their proposal in 2001 (Directive 2001/83/EC later expanded in REGULATION (EC) No 1394/2007; https://www.ema.europa.eu/en/human-regulatory/overview/advanced-therapies/legal-framework-advanced-therapies), GMP have further complicated the development of novel tissue-engineered advanced therapeutic medicinal products (ATMPs). These regulations have proven themselves critical to achieving safer and standardized advanced therapies (Aiuti et al., 2017; Hirsch et al., 2017; Ram-Liebig et al., 2017; Barbagli et al., 2018; Pellegrini et al., 2018; Kueckelhaus et al., 2021). Nevertheless, the resulting complexity and high expenses in the manufacturing process strongly discouraged researchers from exploring the TE area of study. These obstacles have led to the predilection of non-TE approaches, which instead fall under less stringent legislation.

To conclude, tracheal reconstruction stands as a huge challenge and has yet to be achieved. In this review, we summarized the main lessons learned from the clinical and most recent *in vivo* preclinical studies, as well as the new frontiers of tracheal TE. We have highlighted the most common issues that still hinders tracheal reconstruction. In our opinion, a strong correlation between basic science and translational medicine is mandatory; careful and extensive preclinical studies are crucial to tackling all the described aspects. Before embarking on new clinical applications, more work on the preclinical side should be done to prevent patients’ exposure to avoidable life-threatening consequences. Only by addressing these points reconstructive approaches can become a turning point in long-circumferential tracheal defects management and affirm themselves as a milestone in regenerative medicine.

## References

[B1] Abbott O. A. Vanfleit E. Roberto A. E. (1932). THE JOURNAL OF THORACIC SURGERY. Med. J. Aust. 1 (4), 134. 10.5694/j.1326-5377.1932.tb42299.x

[B2] Aiuti A. Roncarolo M. G. Naldini L. (2017). Gene Therapy for ADA‐SCID, the First Marketing Approval of an *Ex Vivo* Gene Therapy in Europe: Paving the Road for the Next Generation of Advanced Therapy Medicinal Products. EMBO Mol. Med. 9 (6), 737–740. 10.15252/emmm.201707573 28396566 PMC5452047

[B3] Alturk A. Bara A. Darwish B. (2020). Since January 2020 Elsevier Has Created a COVID-19 Resource centre with Free Information in English and Mandarin on the Novel Coronavirus COVID- 19 . The COVID-19 Resource centre Is Hosted. Elsevier Connect , the company ’ s public news and information . January.

[B4] Azorin J. F. Bertin F. Martinod E. Laskar M. (2006). Tracheal Replacement with an Aortic Autograft. Eur. J. Cardio-Thoracic Surg. 29 (2), 261–263. 10.1016/j.ejcts.2005.11.026 16388953

[B5] Barbagli G. Akbarov I. Heidenreich A. Zugor V. Olianas R. Aragona M. (2018). Anterior Urethroplasty Using a New Tissue Engineered Oral Mucosa Graft: Surgical Techniques and Outcomes. J. Urol. 200 (2), 448–456. 10.1016/j.juro.2018.02.3102 29601924

[B6] Basil M. C. Katzen J. Engler A. E. Guo M. Herriges M. J. Kathiriya J. J. (2020). The Cellular and Physiological Basis for Lung Repair and Regeneration: Past, Present, and Future. Cell Stem Cell 26 (4), 482–502. 10.1016/j.stem.2020.03.009 32243808 PMC7128675

[B7] Beldholm B. R. Wilson M. K. Gallagher R. M. Caminer D. King M. J. Glanville A. (2003). Reconstruction of the Trachea with a Tubed Radial Forearm Free Flap. J. Thorac. Cardiovasc. Surg. 126 (2), 545–550. 10.1016/S0022-5223(03)00357-X 12928656

[B8] Bertheuil N. Duisit J. Isola N. Lengelé B. Bergeat D. Meunier B. (2021). Perforator-Based Intercostal Artery Muscle Flap: A Novel Approach for the Treatment of Tracheoesophageal or Bronchoesophageal Fistulas. Plast. Reconstr. Surg. 147, 795E–800e. 10.1097/PRS.0000000000007892 33835081

[B9] Blades N. Beattie J. (1986). The Journal of Thoracic Surgery Original Communications TRACHEAL RECONSTRUCTIOK.

[B10] Boazak E. M. Auguste D. T. (2018)., 4. American Chemical Society, 1272–1284. 10.1021/acsbiomaterials.7b00738 Trachea Mechanics for Tissue Engineering Design ACS Biomater. Sci. Eng. Issue 4 33418658

[B11] Bolton W. D. Ben-Or S. Hale A. L. Stephenson J. E. (2017)., 12. Philadelphia, Pa, 137–139. 10.1097/IMI.000000000000034710.1177/155698451701200210 Reconstruction of a Long-Segment Tracheal Defect Using an AlloDerm Conduit Innovations 2 28301367

[B12] Brand-Saberi B. E. M. Schäfer T. (2014). Trachea. Thorac. Surg. Clin. 24 (1), 1–5. 10.1016/j.thorsurg.2013.09.004 24295654

[B13] Cinar U. Halezeroglu S. Okur E. Inanici M. A. Kayaoglu S. (2016). Tracheal Length in Adult Human: The Results of 100 Autopsies. Int. J. Morphol. 34 (1), 232–236. 10.4067/s0717-95022016000100033

[B14] Clagett O. T. Grindlay J. H. Moersch H. J. (1948). Resection of the Trachea. Arch. Surg. 57 (2), 253–266. 10.1001/archsurg.1948.01240020258008 18099766

[B111] Corradini F. Zattoni M. Barbagli G. Bianchi G. Giovanardi M. Serafini C. (2016). Comparative Assessment of Cultures from Oral and Urethral Stem Cells for Urethral Regeneration. Curr. Stem Cell Res. Ther. 11 (8), 643–651. 10.2174/1574888x10666150902094644 26329484

[B15] Cotton B. H. Penido J. R. F. Penido J. R. F. (1952). Resection of the Trachea for Carcinoma. J. Thorac. Surg. 24 (3), 231–245. 10.1016/S0096-5588(20)31076-X 13000917

[B16] Davidson M. B. Mustafa K. Girdwood R. W. (2009). Tracheal Replacement with an Aortic Homograft. Ann. Thorac. Surg. 88 (3), 1006–1008. 10.1016/j.athoracsur.2009.01.044 19699945

[B17] Davis J. D. Wypych T. P. (20212020). Cellular and Functional Heterogeneity of the Airway Epithelium. Mucosal Immunol. 14, 978–990. –13. 10.1038/s41385-020-00370-7 PMC7893625 33608655

[B18] Del Gaudio C. Baiguera S. Ajalloueian F. Bianco A. Macchiarini P. (2014). Are Synthetic Scaffolds Suitable for the Development of Clinical Tissue-Engineered Tubular Organs? J. Biomed. Mater. Res. 102 (7), 2427–2447. 10.1002/jbm.a.34883 23894109

[B19] Delaere P. Van Raemdonck D. (2016). Tracheal Replacement. J. Thorac. Dis. 8 (Suppl. 2), S186–S196. 10.3978/j.issn.2072-1439.2016.01.85 26981270 PMC4775267

[B20] Delaere P. R. Liu Z. Sciot R. Welvaart W. (1996). The Role of Immunosuppression in the Long-Term Survival of Tracheal Allografts. Arch. Otolaryngol. - Head Neck Surg. 122 (11), 1201–1208. 10.1001/archotol.1996.01890230047010 8906055

[B21] Delaere P. R. Liu Z. Y. Hermans R. Sciot R. Feenstra L. (1995). Experimental Tracheal Allograft Revascularization and Transplantation. J. Thorac. Cardiovasc. Surg. 110 (3), 728–737. 10.1016/S0022-5223(95)70105-2 7564440

[B22] Delaere P. R. Van Raemdonck D. (2020). Commentary: The Sobering Truth about Tracheal Regeneration. J. Thorac. Cardiovasc. Surg. 159 (6), 2537–2539. 10.1016/j.jtcvs.2019.10.116 31810651

[B23] Delaere P. R. Van Raemdonck D. (2014). The Trachea: The First Tissue-Engineered Organ? J. Thorac. Cardiovasc. Surg. 147 (4), 1128–1132. 10.1016/j.jtcvs.2013.12.024 24503324

[B24] Delaere P. R. Vranckx J. J. Den Hondt M. (2014). Tracheal Allograft after Withdrawal of Immunosuppressive Therapy. N. Engl. J. Med. 370 (16), 1568–1570. 10.1056/NEJMc1315273 24738689

[B25] Delaere P. R. Vranckx J. J. Meulemans J. Vander Poorten V. Segers K. Van Raemdonck D. (2012). Learning Curve in Tracheal Allotransplantation. Am. J. Transplant. 12 (9), 2538–2545. 10.1111/j.1600-6143.2012.04125.x 22681931

[B26] Delaere P. Van Raemdonck D. Vranckx J. (2019). Tracheal Transplantation. Intensive Care Med. 45 (3), 391–393. 10.1007/s00134-018-5445-9 30430208

[B27] Delaere P. Vranckx J. Verleden G. De Leyn P. Van Raemdonck D. Vranckx J. (2010). Tracheal Allotransplantation after Withdrawal of Immunosuppressive Therapy. N. Engl. J. Med. 362 (7), 138–145. 10.1056/NEJMoa0810653 20071703

[B28] Dharmadhikari S. Best C. A. King N. Henderson M. Johnson J. Breuer C. K. (2019). Mouse Model of Tracheal Replacement with Electrospun Nanofiber Scaffolds. Ann. Otol Rhinol Laryngol. 128 (5), 391–400. 10.1177/0003489419826134 30700095 PMC6530770

[B29] Elliott M. J. Butler C. R. Varanou-Jenkins A. Partington L. Carvalho C. Samuel E. (2017). Tracheal Replacement Therapy with a Stem Cell-Seeded Graft: Lessons from Compassionate Use Application of a GMP-Compliant Tissue-Engineered Medicine. Stem Cell Translational Med. 6 (6), 1458–1464. 10.1002/sctm.16-0443 PMC5689750 28544662

[B30] Elliott M. J. De Coppi P. Speggiorin S. Roebuck D. Butler C. R. Samuel E. (2012). Stem-cell-based, Tissue Engineered Tracheal Replacement in a Child: A 2-year Follow-Up Study. The Lancet 380 (9846), 994–1000. 10.1016/S0140-6736(12)60737-5 PMC4487824 22841419

[B31] Fabre D. Fadel E. Mussot S. Kolb F. Leymarie N. Mercier O. (2015). Autologous Tracheal Replacement for cancerChinese Clinical Oncology. Chin. Clin. Oncol. 4 (Issue 4), 46. 10.3978/j.issn.2304-3865.2015.12.07 26730758

[B32] Fabre D. Kolb F. Fadel E. Mercier O. Mussot S. Le Chevalier T. (2013). Successful Tracheal Replacement in Humans Using Autologous Tissues: An 8-year Experience. Ann. Thorac. Surg. 96 (4), 1146–1155. 10.1016/j.athoracsur.2013.05.073 23998399

[B33] Fabre D. Singhal S. De Montpreville V. Decante B. Mussot S. Chataigner O. (2009). Composite Cervical Skin and Cartilage Flap Provides a Novel Large Airway Substitute after Long-Segment Tracheal Resection. J. Thorac. Cardiovasc. Surg. 138 (1), 32–39. 10.1016/j.jtcvs.2008.11.071 19577053

[B34] Fountain H. (2012). Scientists Make Progress in Tailor-Made Organs. Stockholm: The New York Times.

[B35] Fux T. Österholm C. Themudo R. Simonson O. Grinnemo K.-H. Corbascio M. (2020). Synthetic Tracheal Grafts Seeded with Bone Marrow Cells Fail to Generate Functional Tracheae: First Long-Term Follow-Up Study. J. Thorac. Cardiovasc. Surg. 159 (6), 2525–2537. 10.1016/j.jtcvs.2019.09.185 31859073

[B36] Gallico G. G. (1985). Permanent Coverage of Large Burn Wounds with Autologous Cultured Human Epithelium. Plast. Reconstr. Surg. 76 (5), 812. 10.1097/00006534-198511000-00093 6379456

[B37] Ganesan S. Comstock A. T. Sajjan U. S. (2013). Barrier Function of Airway Tract Epithelium. Tissue Barriers 1, e24997. 10.4161/tisb.24997 24665407 PMC3783221

[B38] Gao B. Jing H. Gao M. Wang S. Fu W. Zhang X. (2019). Long-segmental Tracheal Reconstruction in Rabbits with Pedicled Tissue-Engineered Trachea Based on a 3D-Printed Scaffold. Acta Biomater. 97, 177–186. 10.1016/j.actbio.2019.07.043 31352107

[B39] Gao M. Zhang H. Dong W. Bai J. Gao B. Xia D. (2017). Tissue-engineered Trachea from a 3D-Printed Scaffold Enhances Whole-Segment Tracheal Repair. Sci. Rep. 7 (1), 1–12. 10.1038/s41598-017-05518-3 28701742 PMC5507982

[B40] Genden E. M. Gannon P. J. Deftereos M. Smith S. Urken M. L. (2003). Microvascular Transplantation of Tracheal Allografts in the Canine Model. Ann. Otol Rhinol Laryngol. 112 (4), 307–313. 10.1177/000348940311200404 12731625

[B41] Genden E. M. Gannon P. J. Smith S. Keck N. Deftereos M. Urken M. L. (2002). Microvascular Transfer of Long Tracheal Autograft Segments in the Canine Model. The Laryngoscope 112 (3), 439–444. 10.1097/00005537-200203000-00006 12148850

[B42] Genden E. M. Miles B. A. Harkin T. J. DeMaria S. Kaufman A. J. Mayland E. (2021). Single‐stage Long‐segment Tracheal Transplantation. Am. J. Transpl. 21 (10), 3421–3427. 10.1111/ajt.16752 34236746

[B43] Go T. Jungebluth P. Baiguero S. Asnaghi A. Martorell J. Ostertag H. (2010). Both epithelial Cells and Mesenchymal Stem Cell-Derived Chondrocytes Contribute to the Survival of Tissue-Engineered Airway Transplants in Pigs. J. Thorac. Cardiovasc. Surg. 139 (2), 437–443. 10.1016/j.jtcvs.2009.10.002 19995663

[B44] Goldfarbmuren K. C. Jackson N. D. Sajuthi S. P. Dyjack N. Li K. S. Rios C. L. (2020). Dissecting the Cellular Specificity of Smoking Effects and Reconstructing Lineages in the Human Airway Epithelium. Nat. Commun. 11 (1). 10.1038/s41467-020-16239-z PMC7237663 32427931

[B45] Gonfiotti A. Jaus M. O. Barale D. Baiguera S. Comin C. Lavorini F. (2014). The First Tissue-Engineered Airway Transplantation: 5-year Follow-Up Results. The Lancet 383 (9913), 238–244. 10.1016/S0140-6736(13)62033-4 24161821

[B46] Greaney A. M. Niklason L. E. (2021). The History of Engineered Tracheal Replacements: Interpreting the Past and Guiding the Future. Tissue Eng. B: Rev. 27 (4), 341–352. 10.1089/ten.teb.2020.0238 PMC8390779 33045942

[B47] Grillo H. C. (2002). Tracheal Replacement: A Critical Review. Ann. Thorac. Surg. 73, 1995–2004. 10.1016/s0003-4975(02)03564-6 12078821

[B48] Hamilton N. J. Kanani M. Roebuck D. J. Hewitt R. J. Cetto R. Culme-Seymour E. J. (2015). Tissue-Engineered Tracheal Replacement in a Child: A 4-Year Follow-Up Study. Am. J. Transplant. 15 (10), 2750–2757. 10.1111/ajt.13318 26037782 PMC4737133

[B49] Heijink I. H. Nawijn M. C. Hackett T. L. (2014). Airway Epithelial Barrier Function Regulates the Pathogenesis of Allergic Asthma. Clin. Exp. Allergy 44 (5), 620–630. 10.1111/cea.12296 24612268

[B50] Hirsch T. Rothoeft T. Teig N. Bauer J. W. Pellegrini G. De Rosa L. (2017). Regeneration of the Entire Human Epidermis Using Transgenic Stem Cells. Nature 551 (7680), 327–332. 10.1038/nature24487 29144448 PMC6283270

[B51] Hoffman T. M. Gaynor J. W. Bridges N. D. Paridon S. M. Spray T. L. (2001). Aortic Homograft Interposition for Management of Complete Tracheal Anastomotic Disruption after Heart-Lung Transplantation. J. Thorac. Cardiovasc. Surg. 121 (3), 587–588. 10.1067/mtc.2001.110682 11241097

[B52] Iyer S. Subramaniam N. Vidhyadharan S. Thankappan K. Balasubramanian D. Balasubramanian K. R. (2020). Tracheal Allotransplantation-Lessons Learned. Indian J. Plast. Surg. 53 (2), 306–308. 10.1055/s-0040-1716420 32884201 PMC7458829

[B53] Jungebluth P. Alici E. Baiguera S. Blanc K. Le. Blomberg P. Bozóky B. (2011). Tracheobronchial Transplantation with a Stem-Cell-Seeded Bioartificial Nanocomposite: A Proof-Of-Concept Study. The Lancet 378 (9808), 1997–2004. 10.1016/S0140-6736(11)61715-7 22119609

[B54] Jungebluth P. Haag J. C. Lim M. L. Lemon G. Sjöqvist S. Gustafsson Y. (2019). Retraction Notice to:“Verification of Cell Viability in Bioengineered Tissues and Organs Before Clinical Transplantation ” [BIOMATERIALS (2013) 4057–4067]. Biomaterials 199, 88. 10.1016/j.biomaterials.2019.02.002 30773172

[B55] Jungebluth P. Haag J. C. Lim M. L. Lemon G. Sjöqvist S. Gustafsson Y. (2013). Verification of Cell Viability in Bioengineered Tissues and Organs before Clinical Transplantation. Biomaterials 34 (16), 4057–4067. 10.1016/j.biomaterials.2013.02.057 23473965

[B56] Kim I. G. Park S. A. Lee S. H. Choi J. S. Cho H. Lee S. J. (2020). Transplantation of a 3D-Printed Tracheal Graft Combined with iPS Cell-Derived MSCs and Chondrocytes. Scientific Rep. 10 (1), 1–14. 10.1038/s41598-020-61405-4 PMC7062776 32152475

[B57] Kramish D. Morfit H. M. (1963). The Use of a Teflon Prosthesis to Bridge Complete Sleeve Defects in the Human Trachea. Am. J. Surg. 106 (5), 704–708. 10.1016/0002-9610(63)90388-X 14078720

[B58] Kueckelhaus M. Rothoeft T. De Rosa L. Yeni B. Ohmann T. Maier C. (2021). Transgenic Epidermal Cultures for Junctional Epidermolysis Bullosa - 5-Year Outcomes. New Engl. J. Med. 385 (24), 2264–2270. 10.1056/NEJMoa2108544 34881838

[B59] Lee J. Y. Park J. H. Cho D. W. (2018). Comparison of Tracheal Reconstruction with Allograft, Fresh Xenograft and Artificial Trachea Scaffold in a Rabbit Model. J. Artif. Organs 21 (3), 325–331. 10.1007/s10047-018-1045-2 29752586

[B60] Lee K. S. Yang C. C. (2001). Tracheal Length of Infants under Three Months Old. Ann. Otology, Rhinology Laryngol. 110 (3), 268–270. 10.1177/000348940111000312 11269773

[B61] Levashov Y. U. Yablonsky P. K. Cherny S. M. Orlov S. V. Shafirovsky B. B. Kuznetzov I. M. (1993). One-stage Allotransplantation of Thoracic Segment of the Trachea in a Patient with Idiopathic Fibrosing Mediastinitis and Marked Tracheal Stenosis. Eur. J. Cardio-Thoracic Surg. 7 (7), 383–386. 10.1016/1010-7940(93)90071-I 8373623

[B62] Longmire W. P. (1948). The Repair of Large Defects of the Trachea. Ann. Otology, Rhinology, Laryngol. 57 (3), 875–883. 10.1177/000348944805700322 18885460

[B63] Lovett M. Lee K. Edwards A. Kaplan D. L. (2009). Vascularization Strategies for Tissue Engineering. Tissue Eng. - B: Rev. 15 (3), 353–370. 10.1089/ten.teb.2009.0085 PMC2817665 19496677

[B64] Macchiarini P. Jungebluth P. Go T. Asnaghi M. A. Rees L. E. Cogan T. A. (2008). Clinical Transplantation of a Tissue-Engineered Airway. The Lancet 372 (9655), 2023–2030. 10.1016/S0140-6736(08)61598-6 19022496

[B65] Machino R. Matsumoto K. Taniguchi D. Tsuchiya T. Takeoka Y. Taura Y. (2019). Replacement of Rat Tracheas by Layered, Trachea-like, Scaffold-free Structures of Human Cells Using a Bio-3D Printing System. Adv. Healthc. Mater. 8 (7). 10.1002/adhm.201800983 30632706

[B66] Martínez-Hernández N. J. Mas-Estellés J. Milián-Medina L. Martínez-Ramos C. Cerón-Navarro J. Galbis-Caravajal J. (2021). A Standardised Approach to the Biomechanical Evaluation of Tracheal Grafts. Biomolecules 11 (10), 1–12. 10.3390/biom11101461 PMC8533576 34680094

[B67] Martinod E. Chouahnia K. Radu D. M. Joudiou P. Uzunhan Y. Bensidhoum M. (2018). Feasibility of Bioengineered Tracheal and Bronchial Reconstruction Using Stented Aortic Matrices. JAMA - J. Am. Med. Assoc. 319 (21), 2212–2222. 10.1001/jama.2018.4653 PMC6134437 29800033

[B68] Martinod E. Paquet J. Dutau H. Radu D. M. Bensidhoum M. Abad S. (2017). *In Vivo* Tissue Engineering of Human Airways. Ann. Thorac. Surg. 103 (5), 1631–1640. 10.1016/j.athoracsur.2016.11.027 28109571

[B69] Mattioli F. Marchioni A. Andreani A. Cappiello G. Fermi M. Presutti L. (2021). Post-intubation Tracheal Stenosis in COVID-19 patientsEuropean Archives of Oto-Rhino-Laryngology. Eur. Arch. Otorhinolaryngol. 278 (Issue 3), 847. 10.1007/s00405-020-06394-w 33011955 PMC7532739

[B70] Maughan E. F. Butler C. R. Crowley C. Teoh G. Z. Hondt M. Den. Hamilton N. J. (2017). A Comparison of Tracheal Scaffold Strategies for Pediatric Transplantation in a Rabbit Model. Laryngoscope 127 (12), E449–E457. 10.1002/lary.26611 28776693

[B72] Maurizi E. Adamo D. Magrelli F. M. Galaverni G. Attico E. Merra A. (2021). Regenerative Medicine of Epithelia: Lessons from the Past and Future Goals. Front. Bioeng. Biotechnol. 9 (March). 10.3389/fbioe.2021.652214 PMC8026866 33842447

[B73] Menna C. Andreetti C. Ibrahim M. Ciccone A. M. D’Andrilli A. Maurizi G. (2021). Successful Total Tracheal Replacement by Cryopreserved Aortic Allograft in a Patient Post-COVID-19 Infection. Chest 160 (6), e613–e617. 10.1016/j.chest.2021.08.037 34872673 PMC8640260

[B74] Mercier O. Kolb F. Dartevelle P. G. (2018). Autologous Tracheal Replacement: Surgical Technique and Outcomes. Thorac. Surg. Clin. 28 (3), 347–355. 10.1016/j.thorsurg.2018.05.007 30054072

[B75] Molins L. (2019). Patient Follow-Up after Tissue-Engineered Airway Transplantation. The Lancet 393 (10176), 1099. 10.1016/S0140-6736(19)30485-4 30833040

[B76] Naito H. Tojo T. Kimura M. Dohi Y. Zimmermann W. H. Eschenhagen T. (2011). Engineering Bioartificial Tracheal Tissue Using Hybrid Fibroblastmesenchymal Stem Cell Cultures in Collagen Hydrogels. Interactive Cardiovasc. Thorac. Surg. 12 (2), 156–161. 10.1510/icvts.2010.253559 21098511

[B77] Neville W. E. Bolanowski P. J. P. Kotia G. G. (1990). Clinical Experience with the Silicone Tracheal Prosthesis. J. Thorac. Cardiovasc. Surg. 99 (4), 604–613. 10.1016/s0022-5223(19)36932-6 2319780

[B78] Neville W. E. Bolanowski P. J. Soltanzadeh H. (1976). Prosthetic Reconstruction of the Trachea and Carina. J. Thorac. Cardiovasc. Surg. 72 (4), 525–538. 10.1016/s0022-5223(19)40036-6 966785

[B79] Niermeyer W. L. Rodman C. Li M. M. Chiang T. (2020). Tissue Engineering Applications in Otolaryngology the State of Translation. Laryngoscope Invest. Otolaryngol. 5, 630–648. 10.1002/lio2.416 PMC7444782 32864434

[B80] Olias J. Millán G. da Costa D. (2005). Circumferential Tracheal Reconstruction for the Functional Treatment of Airway Compromise. The Laryngoscope 115 (1), 159–161. 10.1097/01.mlg.0000150688.00635.2b 15630386

[B81] Paternoster D. J. L. Vranckx P. J. J. (2021). State of the Art of Clinical Applications of Tissue Engineering in 2021 10.1089/ten.TEB.2021.0017 34082599

[B82] Pellegrini G. Ardigò D. Milazzo G. Iotti G. Guatelli P. Pelosi D. (2018). Navigating Market Authorization: The Path Holoclar Took to Become the First Stem Cell Product Approved in the European Union. Stem Cell Translational Med. 7 (1), 146–154. 10.1002/sctm.17-0003 PMC5746151 29280318

[B83] Pellegrini G. Rama P. De Luca M. (2011). Vision from the Right Stem. Trends Mol. Med. 17 (1), 1–7. 10.1016/j.molmed.2010.10.003 21075055

[B84] Pepper V. Best C. A. Buckley K. Schwartz C. Onwuka E. King N. (2019). Factors Influencing Poor Outcomes in Synthetic Tissue-Engineered Tracheal Replacement. Otolaryngol. - Head Neck Surg. (United States) 161 (3), 458–467. 10.1177/0194599819844754 PMC7202801 31035858

[B85] Piazza C. Filauro M. Dikkers F. G. Nouraei S. A. R. Sandu K. Sittel C. (2021). Long-term Intubation and High Rate of Tracheostomy in COVID-19 Patients Might Determine an Unprecedented Increase of Airway Stenoses: a Call to Action from the European Laryngological Society. Eur. Arch. Oto-Rhino-Laryngology 278 (1), 1–7. 10.1007/s00405-020-06112-6 PMC7275663 32506145

[B86] Ram-Liebig G. Barbagli G. Heidenreich A. Fahlenkamp D. Romano G. Rebmann U. (2017). Results of Use of Tissue-Engineered Autologous Oral Mucosa Graft for Urethral Reconstruction: A Multicenter, Prospective, Observational Trial. EBioMedicine 23, 185–192. 10.1016/j.ebiom.2017.08.014 28827035 PMC5605371

[B87] Randhawa S. K. Patterson G. A. (2021). Single-stage Tracheal Transplantation—From Bench to Bedside. Am. J. Transplant. 21 (10), 3223–3224. 10.1111/ajt.16776 34331841

[B88] Rehmani S. S. Al-Ayoubi A. M. Ayub A. Barsky M. Lewis E. Flores R. (2017). Three-Dimensional-Printed Bioengineered Tracheal Grafts: Preclinical Results and Potential for Human Use. Ann. Thorac. Surg. 104 (3), 998–1004. 10.1016/j.athoracsur.2017.03.051 28610885

[B110] Rose K. G. Sesterhenn K. Wustrow F. (1979). Tracheal Allotransplantation in Man. Lancet 1 (8113), 433. 10.1016/s0140-6736(79)90902-4 84276

[B89] Ruiz García S. Deprez M. Lebrigand K. Cavard A. Paquet A. Arguel M.-J. (2019)., 146. Cambridge. 10.1242/dev.177428 Novel Dynamics of Human Mucociliary Differentiation Revealed by Single-Cell RNA Sequencing of Nasal Epithelial Cultures Development Issue 20 PMC6826037 31558434

[B90] Sakaguchi Y. Sato T. Muranishi Y. Yutaka Y. Komatsu T. Omori K. (2018). Development of a Novel Tissue-Engineered Nitinol Frame Artificial Trachea with Native-like Physical Characteristics. J. Thorac. Cardiovasc. Surg. 156 (3), 1264–1272. 10.1016/j.jtcvs.2018.04.073 29779644

[B91] Salassa J. R. Pearson B. W. Payne W. S. (1977). Gross and Microscopical Blood Supply of the Trachea. Ann. Thorac. Surg. 24 (2), 100–107. 10.1016/S0003-4975(10)63716-2 327958

[B92] Schneider P. Schirren J. Muley T. Vogt-Moykopf I. (2001). Primary Tracheal Tumors: Experience with 14 Resected Patients. Eur. J. Cardio-Thoracic Surg. 20 (1), 12–18. 10.1016/S1010-7940(01)00732-1 11423267

[B93] Shafiee A. Atala A. (2017). Tissue Engineering: Toward a New Era of Medicine. Annu. Rev. MedicineAnnu Rev Med 68, 29–40. 10.1146/annurev-med-102715-092331 27732788

[B94] Soriano L. Khalid T. Whelan D. O’Huallachain N. Redmond K. C. O’Brien F. J. (2021). Development and Clinical Translation of Tubular Constructs for Tracheal Tissue Engineering: a Review. Eur. Respir. Rev. 30 (162), 210154. 10.1183/16000617.0154-2021 34750116 PMC9488721

[B95] Spaggiari L. Calabrese L. S. D’Aiuto M. Veronesi G. Galetta D. Venturino M. (2005). Successful Subtotal Tracheal Replacement (Using a Skin/omental Graft) for Dehiscence after a Resection for Thyroid Cancer. J. Thorac. Cardiovasc. Surg. 129 (6), 1455–1456. 10.1016/j.jtcvs.2004.11.010 15942602

[B96] Tan Q. Liu R. Chen X. Wu J. Pan Y. Lu S. (2017). Clinic Application of Tissue Engineered Bronchus for Lung Cancer Treatment. J. Thorac. Dis. 9 (1), 22–29. 10.21037/jtd.2017.01.50 28203403 PMC5303100

[B97] Taniguchi D. Matsumoto K. Tsuchiya T. MacHino R. Takeoka Y. Elgalad A. (2018). Scaffold-free Trachea Regeneration by Tissue Engineering with bio-3D Printing. Interactive Cardiovasc. Thorac. Surg. 26 (5), 745–752. 10.1093/icvts/ivx444 29346562

[B98] Teng Z. Trabelsi O. Ochoa I. He J. Gillard J. H. Doblare M. (2012). Anisotropic Material Behaviours of Soft Tissues in Human Trachea: An Experimental Study. J. Biomech. 45 (9), 1717–1723. 10.1016/j.jbiomech.2012.04.002 22534565

[B99] The Lancet Editors (2018). Retraction—Tracheobronchial Transplantation with a Stem-Cell-Seeded Bioartificial Nanocomposite: a Proof-Of-Concept Study. The Lancet 392 (10141), 11. 10.1016/S0140-6736(18)31558-7 30047380

[B100] The Lancet (2018). The Final Verdict on Paolo Macchiarini: Guilty of Misconduct. The Lancet 392 (10141), 2. 10.1016/S0140-6736(18)31484-3 30047386

[B101] Toomes H. Mickisch G. Vogt-Moykopf I. (1985). Experiences with Prosthetic Reconstruction of the Trachea and Bifurcation. Thorax 40 (1), 32–37. 10.1136/thx.40.1.32 3969653 PMC459974

[B102] Udelsman B. Mathisen D. J. Ott H. C. (2018). A Reassessment of Tracheal Substitutes-A Systematic Review. Ann. Cardiothorac. Surg. 7 (2), 175–182. 10.21037/acs.2018.01.17 29707495 PMC5900077

[B103] Vranckx J. J. Hondt M. Den. (2017). Tissue Engineering and Surgery: From Translational Studies to Human Trials. Innovative Surg. Sci. 2 (4), 189–202. 10.1515/iss-2017-0011 PMC6754028 31579752

[B104] Wurtz A. Porte H. Conti M. Desbordes J. Copin M. C. Azorin J. (20061940). Tracheal Replacement with Aortic Allografts. New Engl. J. Med. 355 (18), 1938. 10.1056/NEJMc066336 17079776

[B105] Wurtz A. Porte H. Conti M. Dusson C. Desbordes J. Copin M.-C. (2010). Surgical Technique and Results of Tracheal and Carinal Replacement with Aortic Allografts for Salivary Gland-type Carcinoma. J. Thorac. Cardiovasc. Surg. 140 (2), 387–393. e2. 10.1016/j.jtcvs.2010.01.043 20381819

[B106] Yu P. Clayman G. L. Walsh G. L. (2006). Human Tracheal Reconstruction with a Composite Radial Forearm Free Flap and Prosthesis. Ann. Thorac. Surg. 81 (2), 714–716. 10.1016/j.athoracsur.2004.12.009 16427881

[B107] Zaragosi L. E. Deprez M. Barbry P. (2020). Using Single-Cell RNA Sequencing to Unravel Cell Lineage Relationships in the Respiratory Tract. Biochem. Soc. Trans. 48 (1), 327–336. 10.1042/BST20191010 31922198

[B108] Zhang S. Liu Z. (2015). Airway Reconstruction with Autologous Pulmonary Tissue Flap and an Elastic Metallic Stent. World J. Surg. 39 (8), 1981–1985. 10.1007/s00268-015-3066-9 25900710

[B109] Zhao L. Sundaram S. Le A. V. Huang A. H. Zhang J. Hatachi G. (2016). Engineered Tissue-Stent Biocomposites as Tracheal Replacements. Tissue Eng. - A 22 (17–18), 1086–1097. 10.1089/ten.tea.2016.0132 PMC5312617 27520928

